# Anterior Maxillary Mesiodens Extraction in a Young Adult Male: A Case Report

**DOI:** 10.7759/cureus.64208

**Published:** 2024-07-10

**Authors:** Gaurav Hemnani, Neha Pankey, Manoj Chandak, Ramakrishna Yeluri, Priyanka D Sontakke, Harshita S Dhanrajani

**Affiliations:** 1 Dentistry, Sharad Pawar Dental College and Hospital, Datta Meghe Institute of Higher Education and Research, Wardha, IND; 2 Pediatric and Preventive Dentistry, Sharad Pawar Dental College and Hospital, Datta Meghe Institute of Higher Education and Research, Wardha, IND; 3 Conservative Dentistry and Endodontics, Sharad Pawar Dental College and Hospital, Datta Meghe Institute of Higher Education and Research, Wardha, IND; 4 Public Health Dentistry, Sharad Pawar Dental College and Hospital, Datta Meghe Institute of Higher Education and Research, Wardha, IND

**Keywords:** palatally placed, extraction, maxillary central incisor, mesiodens, supernumerary teeth

## Abstract

Mesiodens are classified as supernumerary teeth located in the maxilla, specifically in the palatal region between the central incisors. The prevalence of this condition varies between 0.15% and 1.9% among individuals. The presence of mesiodens can potentially affect the normal growth and development of teeth, leading to delayed eruption of permanent teeth, displacement or rotation of adjacent teeth, root resorption, and infectious pulpitis. This report discusses the case of a 14-year-old male patient who presented with a palatally placed mesiodens, which was completely erupted in the oral cavity. The patient underwent extraction for the removal of the mesiodens, and the procedure was successful with no complications reported during or after surgery. Early identification and surgical removal of mesiodens are crucial to preventing potential orthodontic and functional complications. This case highlights the importance of regular dental checkups for diagnosing supernumerary teeth. Timely intervention can lead to favorable outcomes, as demonstrated in this patient.

## Introduction

Supernumerary teeth are additional teeth that develop in the mouth and can affect the eruption and alignment of normal teeth. They are more common in the maxilla and can be found in 5.7% to 18.7% of mixed dentition cases. The various types of supernumerary teeth include mesiodens, paramolars, and distomolars, with mesiodens being the most frequently observed [1]. Mesiodens are categorized into two groups: rudimentary, which occurs in permanent dentition, and supplementary, which occurs in deciduous dentition. They are further classified as tuberculate, conical, or molariform mesiodens according to morphology [2,3].

Supplementary mesiodens mimic natural teeth in terms of size and shape, while rudimentary mesiodens are characterized by their unusual morphology and reduced dimensions [2]. Conical mesiodens are often peg-shaped and are usually found in the palatal region, likely to displace the emerging permanent central incisors and result in diastema [4-6]. However, they may exhibit inversion, with the crown positioned upwards, reducing the probability of oral cavity eruption; occasionally, inverted conical mesiodens might emerge into the nasal cavity [7]. Tuberculate mesiodens have a barrel-like shape, feature numerous tubercles, and display irregular root development. The molariform type presents with a crown-like premolar and a completely developed root. It is the rarest of all types [2].

Mesiodens often lead to delayed eruption of permanent teeth, midline displacement, tooth rotation, and root resorption of adjacent teeth, among other complications. Supernumerary teeth are typically extracted at 5-10 years to prevent such complications and avoid the need for orthodontic treatment, which can be lengthy and bothersome. [8]. The mesiodens, typically located beside the maxillary central incisors, are the most prevalent type of supernumerary tooth [9]. Almost all surgeons opt for the extraction of mesiodens as the first line of treatment. If the tooth is impacted, a palatal approach is most preferred, as they mostly erupt palatally [9].

## Case presentation

The 14-year-old male patient presented to the Department of Preventive and Pediatric Dentistry with a primary concern of pain over the temporomandibular joint and behind the right ear. He described the pain as dull and throbbing, which was persistent and did not subside. The pain had been present for the past two weeks. The patient provided no history of trauma. There was no associated swelling, fever, or discharge. He reported that the pain worsened while chewing hard food. The patient had tried over-the-counter pain medications such as ibuprofen 200 mg, which provided temporary relief but did not alleviate the pain completely. He denied any other dental or oral health issues in the past. He did not report any systemic conditions, and the patient's parents provided no significant family history.

**Figure 1:**
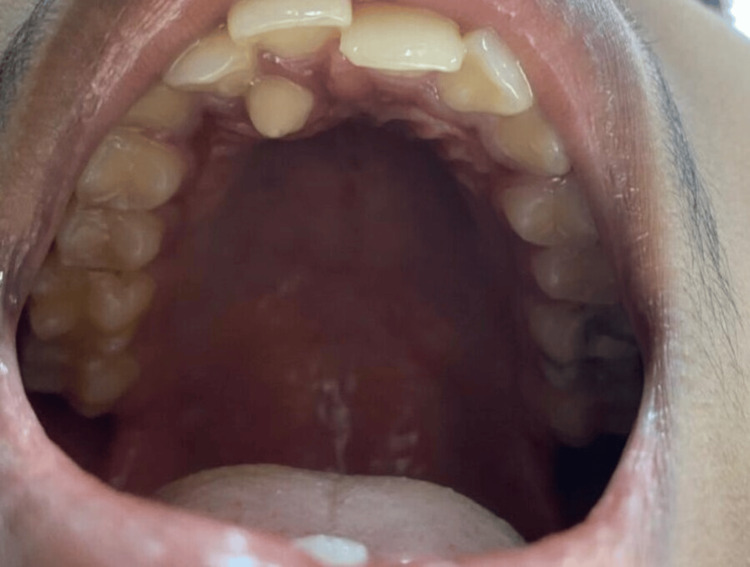
Clinical photograph of the maxilla showing the mesiodens

An intraoral clinical examination revealed the presence of a supernumerary tooth, which was diagnosed as a mesiodens, and it was positioned between the maxillary right permanent central and lateral incisors on the palatal aspect. Due to the presence of the mesiodens, there was premature contact between the teeth, which resulted in an imbalanced occlusion and a change in the curve of Spee. There was crowding present over the maxillary anterior region, and the patient was unable to completely close the mouth as the lower incisors were encountering the supernumerary tooth present, which also resulted in referred pain in the temporomandibular joint. The maxillary right permanent central incisor was more inclined labially, which resulted in an increased overjet (2 mm greater than normal). Figure 1 depicts an intraoral clinical photograph of the maxillary anterior region, showing the mesiodens.

**Figure 2:**
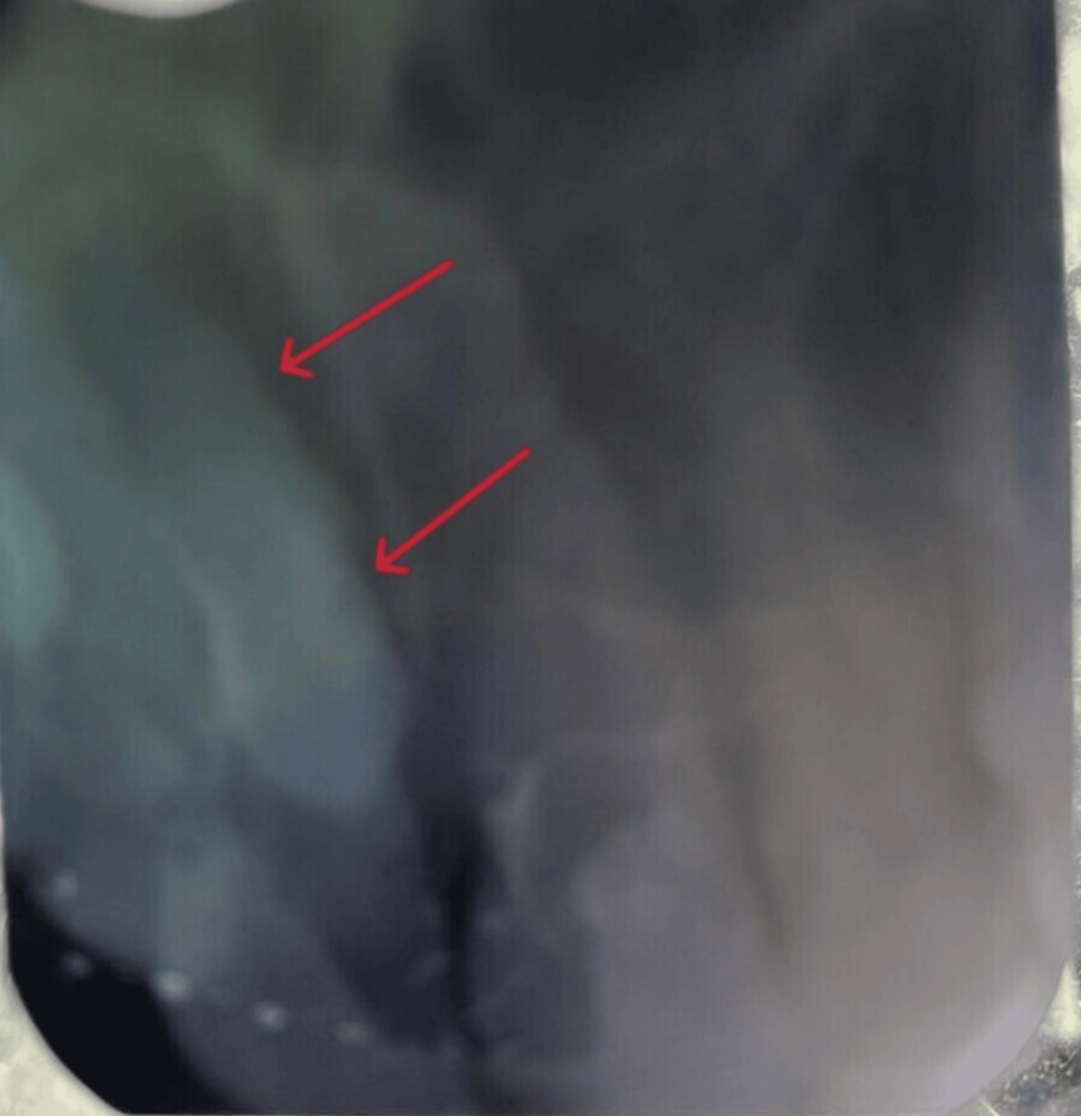
Intraoral radiograph of the maxillary anterior region showing the mesiodens (red arrows)

An intraoral periapical radiograph was recommended to locate the exact position of the mesiodens in the maxillary anterior region. The fully visible teeth were 11, 21, and 12, and the teeth seen partially were 22 and 13. Additionally, a mesiodens association with tooth 11 was observed. The mesiodens was classified as a rudimentary conical mesiodens in the palatal aspect and in proximity with tooth 11. It did not cross the midline of the palate. Figure 2 shows an intraoral periapical radiograph of the maxillary anterior region.

After considering the radiographic findings and correlating them with the clinical features and chief complaint, a diagnosis of supernumerary teeth was made. The mesiodens interfered with the occlusion, leading to premature contact between the teeth and resulting in pain in the temporomandibular joint region.

Treatment

**Figure 3:**
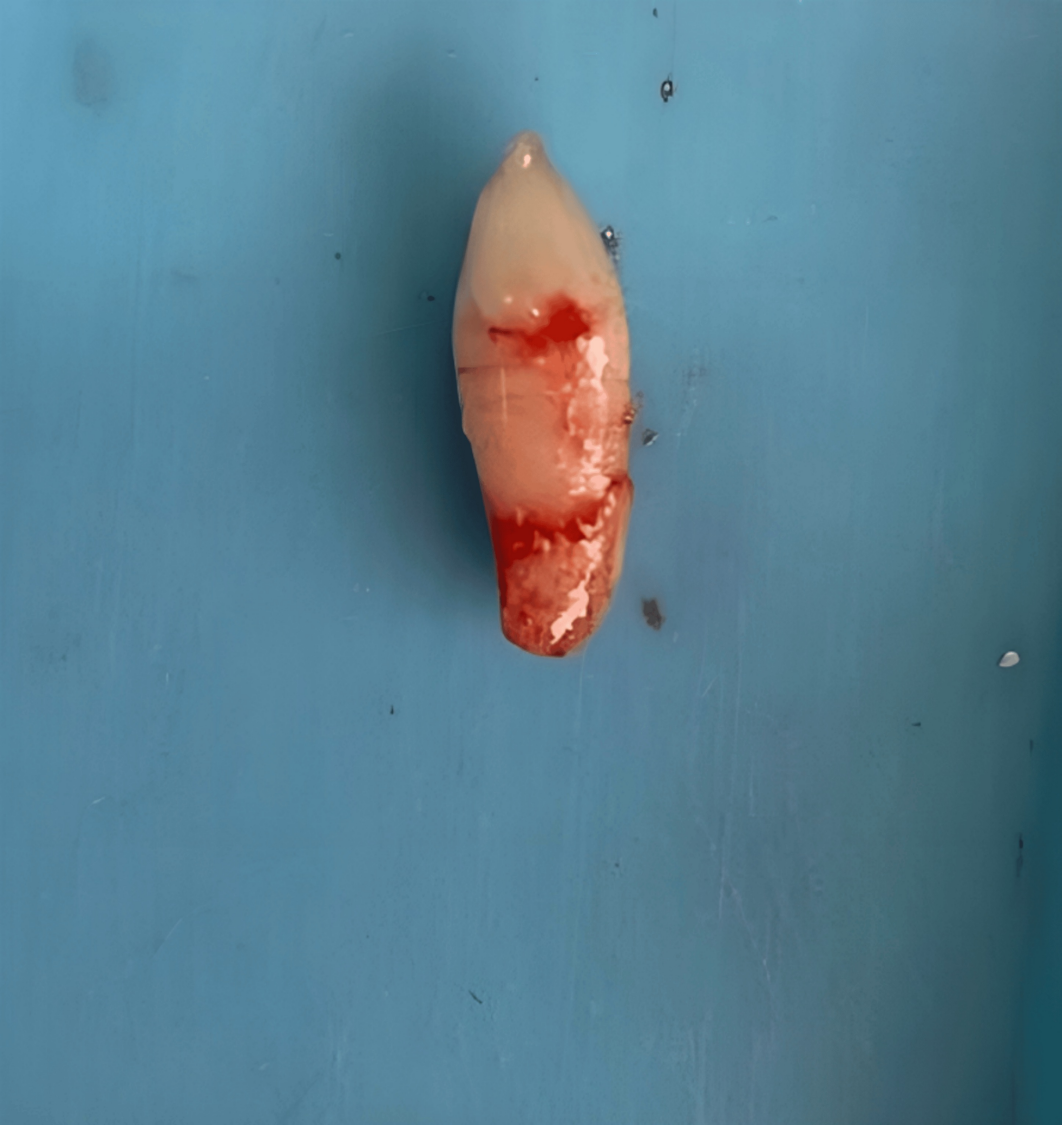
The extracted supernumerary tooth (mesiodens)

**Figure 4:**
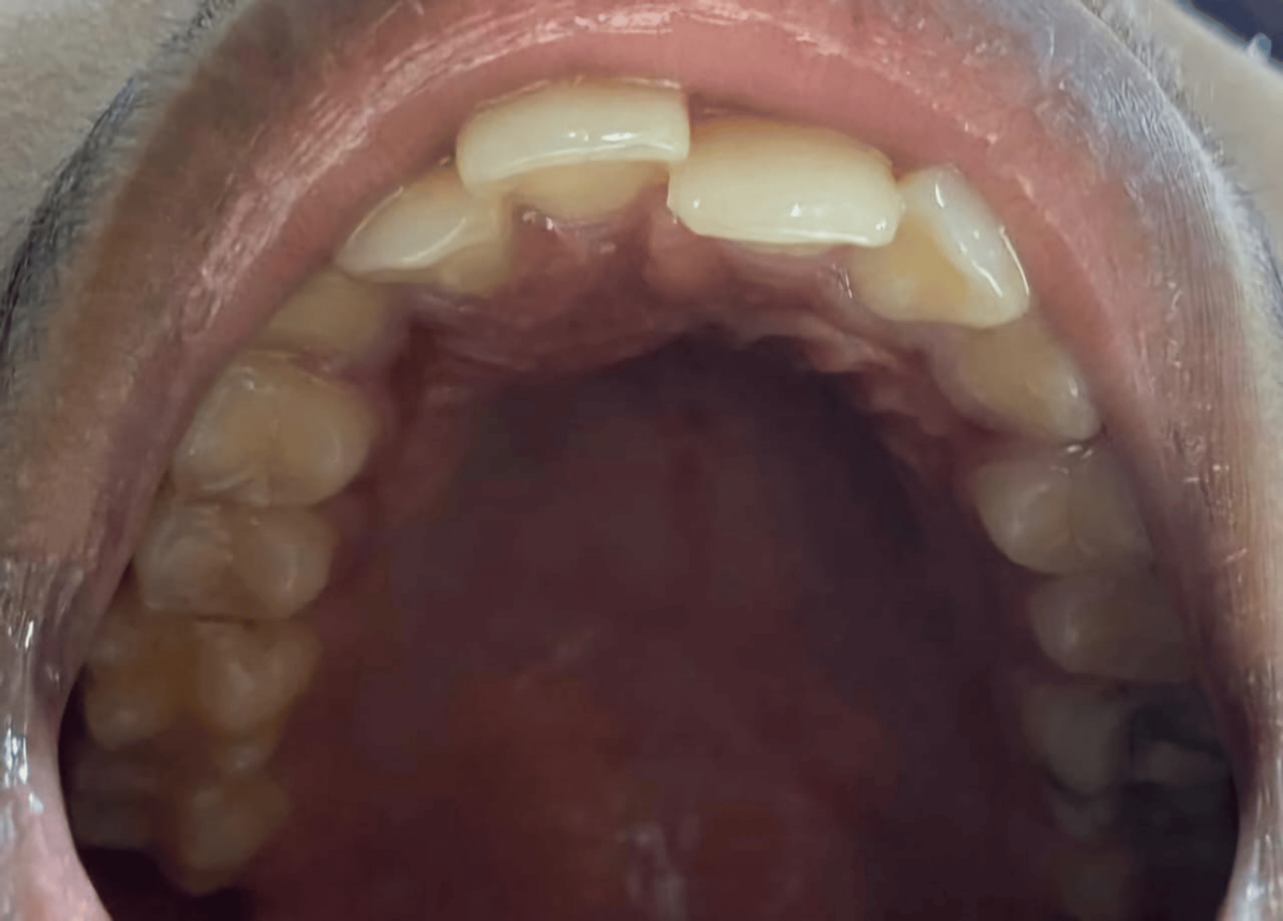
Follow-up intraoral clinical photograph after 14 days

Based on all the clinical findings and examinations, an extraction of the mesiodens was planned. The patient's parents were informed about the treatment plan and all the steps involved in the procedure. Signed informed consent was obtained from the parents. The patient was not allergic to any drugs or medication known to him. After administering local anesthetic through injection and achieving all subjective and objective signs and symptoms, extraction of the mesiodens was performed. Hemostasis was achieved, and a pressure pack was given. Post-procedural instructions were given to the patient, such as removal of cotton placed in the extraction site after one hour, and if bleeding persists, visit the dental hospital to consult the doctor. The patient was advised on the application of an ice pack to control the bleeding and also to avoid hot food for two to three days. The patient was recalled for a follow-up after 14 days, where it was observed that the patient did not experience pain or difficulty in mastication or closing their mouth. Figure 3 shows the extracted mesiodens, and Figure 4 shows a postoperative clinical photograph of the maxillary anterior region after 14 days.

## Discussion

The literature documents cases where an individual can exhibit various isolated dental anomalies, such as supernumerary teeth, missing teeth, alterations in shape or size, dental impaction, discoloration, or structural irregularities [10]. We presented this case to show the incidence of mesiodens in young children and its various associated complications. Various types of supernumerary teeth occurring in the oral cavity are mesiodens, paramolars, and distomolars [11,12]. They can lead to complications and may necessitate prompt treatment. Various hypotheses have been put forward describing the cause of supernumerary teeth. The "phylogenetic theory" posits that the emergence of supernumerary teeth is due to the reappearance of traits from extinct ancestors. The dental lamina splits into various parts, resulting in the formation of a supernumerary tooth, according to the tooth germ dichotomy theory. The "hyperactivity of dental lamina" theory suggests that supernumerary teeth develop because of the overgrowth of remnants of dental lamina [13]. Alarcon et al. (2022) reported three cases of mesiodens, which indicated that heredity is involved in the etiology of supernumerary teeth [14].

Mesiodens can be fully erupted in the oral cavity, as seen in our case, or they can be impacted/unerupted as well, which are accidentally found in routine dental checkups or as an accidental finding in radiographs. In 2023, Kimura and colleagues categorized mesiodens based on their vertical and horizontal positions, recommending the nasal floor approach for severely impacted mesiodens located anterior to the nasopalatine duct, particularly when the angle between the nasal floor line and the mesiodens axis is less than 90° [15]. Yamamoto and Hanazawa [16] described three cases of mesiodens with angles ranging from 85° to 106° between the palatal plane and the mesiodens axis and distances of 10-13 mm from the ANS, advocating for the nasal floor approach when the angle is below 90°. Among impacted mesiodens, 80% are found on the palatal side, 6% on the labial side, and 14% between the apex of two permanent central incisors [17,18].

In this case, the reported mesiodens was placed palatally, and due to this, the patient had difficulty with mastication and an inability to close the mouth due to premature contact. Various other complications encountered in this case were crowding, a diastema, premature contact of teeth, imbalanced occlusion, a change in the curve of Spee, and increased overjet [19,20]. Due to improper occlusion and premature contact of teeth, the patient had severe pain in the temporomandibular joint region, which also radiated behind the right ear. The overjet was also increased in this case, which led to unappealing esthetics [21]. As the mesiodens fully erupted into the oral cavity, routine extraction was performed, which was uneventful. The patient may have to undergo orthodontic treatment to correct increased overjet and crowding. After a 14-day follow-up, it was observed that the patient had no pain and had no more difficulty with mastication as well as in the closure of the mouth.

## Conclusions

Typically, the discovery of mesiodens occurs after complications arise, often noticeable due to patient complaints or during clinical and radiographic evaluations. Hence, prompt and early detection is crucial in preventing these issues. This study urges dentists to proactively identify mesiodens by reviewing clinical or family history, performing routine radiographic exams, and conducting thorough clinical examinations. This proactive approach enables early intervention, even in patients who do not exhibit symptoms, helps prevent complications, and allows for treatment planning tailored to each case's specific location and anatomical context.
